# Rapid tests and urine sampling techniques for the diagnosis of urinary tract infection (UTI) in children under five years: a systematic review

**DOI:** 10.1186/1471-2431-5-4

**Published:** 2005-04-05

**Authors:** Penny Whiting, Marie Westwood, Ian Watt, Julie Cooper, Jos Kleijnen

**Affiliations:** 1 MRC Health Services Research Collaboration, University of Bristol, England, UK; 2 Centre for Reviews and Dissemination, University of York, England, UK; 3 Department of Health Sciences, University of York, England, UK; 4 Department of Radiology, York District Hospital, England, UK

## Abstract

**Background:**

Urinary tract infection (UTI) is one of the most common sources of infection in children under five. Prompt diagnosis and treatment is important to reduce the risk of renal scarring. Rapid, cost-effective, methods of UTI diagnosis are required as an alternative to culture.

**Methods:**

We conducted a systematic review to determine the diagnostic accuracy of rapid tests for detecting UTI in children under five years of age.

**Results:**

The evidence supports the use of dipstick positive for both leukocyte esterase and nitrite (pooled LR+ = 28.2, 95% CI: 17.3, 46.0) or microscopy positive for both pyuria and bacteriuria (pooled LR+ = 37.0, 95% CI: 11.0, 125.9) to rule in UTI. Similarly dipstick negative for both LE and nitrite (Pooled LR- = 0.20, 95% CI: 0.16, 0.26) or microscopy negative for both pyuria and bacteriuria (Pooled LR- = 0.11, 95% CI: 0.05, 0.23) can be used to rule out UTI. A test for glucose showed promise in potty-trained children. However, all studies were over 30 years old. Further evaluation of this test may be useful.

**Conclusion:**

Dipstick negative for both LE and nitrite or microscopic analysis negative for both pyuria and bacteriuria of a clean voided urine, bag, or nappy/pad specimen may reasonably be used to rule out UTI. These patients can then reasonably be excluded from further investigation, without the need for confirmatory culture. Similarly, combinations of positive tests could be used to rule in UTI, and trigger further investigation.

## Background

Urinary tract infection (UTI) is one of the most common sources of infection in children under 5. In a small proportion of children UTI may lead to renal scarring [1,2]. This outcome of infection is of concern as it is associated with significant future complications and ultimately with end stage renal disease[3]. Prompt diagnosis and treatment is therefore important to reduce the risk of future scarring.

Clinical history and examination is the first step in any diagnosis and is the means of identifying children with suspected UTI. Elements of the clinical examination have also been evaluated as diagnostic tests for UTI but there is little data available on these. Urine tests are commonly used for the diagnosis of UTI.

The reference standard for the diagnosis of UTI in children is considered to be any bacterial growth on a culture of urine obtained by suprapubic aspiration[4]. Culture has the disadvantage of taking at least 48 hours to give a result. More rapid methods of UTI diagnosis are therefore desirable. The most widely used rapid tests are dipsticks. Analytes commonly tested by dipsticks include leukocyte esterase, nitrite, blood and protein[4]. Dipstick tests have the advantage of being quick and easy to perform and can be carried out in primary care giving an immediate result. Microscopic examination of urine samples for leukocytes or bacteria [4] is considerably more time consuming and labour intensive than the dipstick method[5]. However, unlike culture, it can be used to give results within the primary care setting. An uncontaminated sample is necessary to reach an accurate diagnosis. Obtaining this is a particular issue when investigating young children. Table 1 presents a summary of the advantages and disadvantages of these tests.

**Table 1:** Details of tests evaluated in the review

**Test**	**Details**	**Advantages**	**Disadvantages**
**Urine sampling**			

*Suprapubic aspiration (SPA)*	Needle attached to syringe inserted through lower abdomen into bladder.	Least risk of contamination	Invasive
*Transurethral catheterisation*	Catheter inserted through the urethra into the bladder.	Less invasive than SPA	Invasive, causes pain and distress to child
*Clean voided urine (CVU)*	Midstream sample collected in sterile container.	Non-invasive, easy to obtain	Difficult in younger children
*Urine bags*	Bag applied to perineum.	Suitable for babies and infants	Risk of contamination
*Urine pads*	Absorbent pad placed in nappy.		

**Dipstick**			

Nitrite	Gram-negative bacteria reduce dietary nitrate to nitrites.	Very easy and quick to perform, relatively cheap	Less accurate than culture
*Leukocyte esterase (LE) Glucose*	Leukocyte esterase is an enzyme that suggests the presence of leukocytes. Normal urine contains small amount of glucose. Bacteria metabolise glucose and so this test tests for the absence of glucose. Requires morning fasting urine specimen.		Not commercially available, not suitable for non-potty trained children

**Microscopy**			

Pyuria	Urine examined through microscope for presence of white blood cells. Samples may be centrifuged before examination	Quicker than culture	More time consuming than dipstick, more expensive than dipstick and culture
*Bacteriuria*	Urine examined for presence of bacteria. Urine may be Gram-stained.		

**Culture**			

*Standard Culture*	Reference standard test for UTI. Involves streaking urine on enrichment and selective media.	Very accurate	Time consuming: takes 48 hours to give a result, has to be performed in the laboratory

A wide range of other tests have been evaluated for the diagnosis of UTI. These include dipslide and rapid culture methods, colorimetric tests, headspace gas analysis, impedance, bio- and chemical luminescence, immunologic tests (e.g. ELISA), enzyme tests, bacterial oxygen consumption, and turbidimetry. However, these are not in widespread use and will not be discussed in this paper.

This review aims to determine the diagnostic accuracy of dipstick and microscopy, and different methods of urine sampling, for detecting UTI in children under five years of age. Two previous reviews have addressed a similar objective[6,7]. These were published over 2 years ago and did not assess urine sampling. They also included fewer studies (48 and 26 compared to 70), possibly as a result of less extensive literature searches and tighter inclusion criteria, than this review. This review therefore presents the most up to date and extensive systematic review of the topic area.

## Methods

We searched 16 electronic databases from inception to between October 2002 and February 2003. Update searches were conducted in May 2004. To identify additional published and unpublished studies we searched the internet, hand searched 12 key journals, screened reference lists of included papers and contacted experts in the field.

We did not apply any language restrictions. Full details of the search strategy will be reported elsewhere[8].

Studies had to meet the following criteria to be included in the review:

*Study design*: diagnostic cohort (single sample) studies

*Population*: at least some children aged <5 years with suspected UTI

*Index tests: *microscopy or dipstick tests used to diagnose UTI or an evaluation of urine sampling methods.

*Reference standard: *culture or culture combined with other tests

*Outcome measures: *sufficient information to construct a 2 × 2 table

Two reviewers independently screened titles and abstracts for relevance, we resolved disagreements by consensus. One reviewer performed inclusion assessment; data extraction and quality assessment and a second reviewer checked this. We extracted 2 × 2 data and used this to calculate measures of diagnostic performance. We used QUADAS to assess study quality[9]. Individual QUADAS items were used to investigate heterogeneity and to present a detailed assessment of quality to the reader.

For each test, or test combination, we calculated the range in sensitivity, specificity, positive (LR+) and negative (LR-) likelihood ratios, and diagnostic odds ratios (DOR). We selected likelihood ratios as the measure of test performance for further analysis as these measures are easier to interpret than sensitivity and specificity [10]. For tests investigated in more than two studies, we used random effects models to pool positive and negative likelihood ratios [11]. Where studies presented more than one estimate of test performance for the same test, for example at different cut-off points or for different patient subgroups, we only included one estimate in the pooled analysis. We aimed to select the data set most similar to the estimates provided by the other studies in terms of population, test manufacturer or population. Heterogeneity of likelihood ratios was investigated using the Q statistic [12] and through visual examination of forest plots of study results [13].

We presented individual studies results graphically by plotting estimates of sensitivity and specificity in receiver operating characteristic (ROC) space. Where sufficient data were available, we used regression analysis to investigate heterogeneity. We extended the summary ROC (sROC) model [14], estimated by regressing D (log DOR) against S (logit true positive rate – logit false positive rate), weighted according to sample size, to include covariates relating to patient age (<2 years, <5 years, <12 years and <18 years), geographic region and each of the 14 QUADAS items. In addition, for microscopy for pyuria and bacteriuria a variable on whether the sample was centrifuged was included, and for microscopy for bacteriuria a variable for Gram stain was included.

## Results

**Figure 1:**
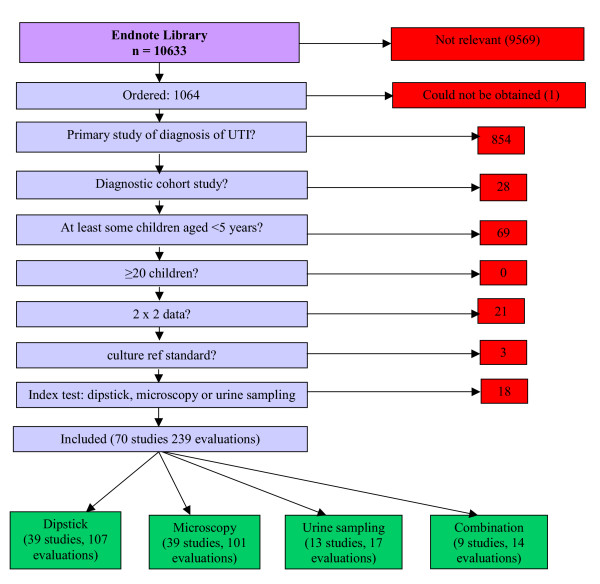
Flow chart of studies through review process.

The literature searches identified over 10 000 references of which 70 studies were included. Figure 1 shows the flow of studies through the review process. A summary of the results of all 70 studies included in the review is provided [see Additional file 1].

### Quality

**Figure 2:**
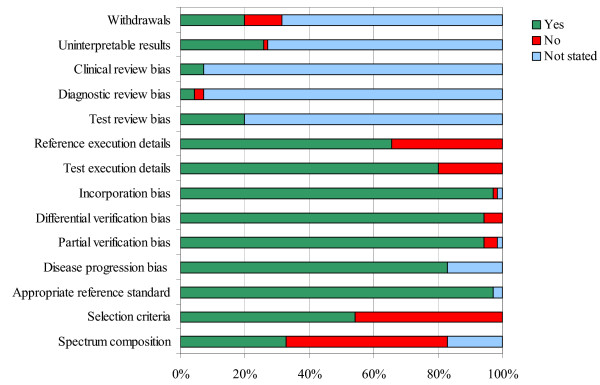
Results of the quality assessment.

The median number of the 14 QUADAS items fulfilled was 8 (range 5–13). The main limitation with the studies was the failure to include an appropriate patient spectrum (<40%) or to report inclusion criteria. Studies also failed to report sufficient details to judge whether clinical review bias (the availability of clinical information to the person interpreting the test results), diagnostic review bias (the availability of the results of the index test to the person interpreting the reference standard) and test review bias (the availability of the results of the reference standard to the person interpreting the index test) were avoided. Withdrawals and handling of uninterpretable results were also poorly reported. Figure 2 illustrates the number of studies that answered "yes", "no" and "not stated" to each of the 14 QUADAS items. A summary of the results of the quality assessment for each study is provided [see Additional file 2].

### Urine sampling

Thirteen studies, with a total of 17 different test evaluations, compared the diagnostic accuracy of different methods obtaining urine for testing [15-27]. These studies compared the results of culture from urine obtained by different sampling methods. Five studies reporting seven data sets assessed the diagnostic accuracy of a clean voided urine (CVU) sample, using a supra-pubic aspiration (SPA) urine sample as the reference standard [15-19]. When both samples were cultured the agreement between the two sampling methods was good. There was considerable heterogeneity in positive likelihood ratios (p < 0.0001). However, the negative likelihood ratios were statistically homogeneous (p = 0.531). The pooled positive likelihood ratio for a CVU sample was 8.8 (95% CI: 2.6, 29.6) and the pooled negative likelihood ratio was 0.23 (95% CI: 0.18, 0.30). Overall, there were insufficient data to draw any conclusions regarding the appropriateness of using urine samples obtained from bags (4 studies)[16,20,21,27] or pads/nappies (4 studies) [22-25].

### Dipstick tests

A total of 39 studies reporting 107 data sets evaluated dipstick tests for the diagnosis of UTI [28-66]. These studies assessed the utility of dipstick tests for nitrite, leukocyte esterase (LE), protein, glucose and blood, alone and in combination. Table 2 summarises the results of these studies.

**Table 2:** Summary of results for studies of dipstick tests

**Dipstick positive for:**	**Number of studies**	**Range in LR+**	Pooled LR+ (95 % CI)*	**Range in LR-**	Pooled LR- (95 % CI)*
Nitrite	23	2.5 – 439.6	15.9 (10.7, 23.7)	0.12 – 0.86	0.51 (0.43, 0.60)
LE	14	2.6 – 32.2	5.5 (4.1, 7.3)	0.02 – 0.66	0.26 (0.18, 0.36)
Nitrite or LE	15	3.0 – 32.2	6.1 (4.3, 8.6)	0.03 – 0.39	0.20 (0.16, 0.26)
Nitrite and LE	9	6.3 – 197.1	28.2 (17.3–46.0)	0.07 – 0.86	0.37 (0.26, 0.52)
Glucose	4	25.2 – 156.1	66.3 (20.0, 219.6)	0.02 – 0.38	0.07 (0.01, 0.83)
Protein	2	1.7 & 1.8	na	0.78 & 0.96	na
Blood	1	2.3	na	0.84	na
LE and protein	1	17.4	na	0.12	na
Nitrite, blood, or protein	1	2.7	na	0.28	na
Nitrite, blood, or LE	1	1.3	na	0.50	na
Nitite, blood and LE	1	3.5	na	0.19	na
Nitrite, LE and protein	2	3.1 & 69.2	na	0.05 & 0.17	na
Nitrite, LE, or protein	1	1.9	na	0.05	na

Nitrite, LE, protein, or blood	1	8.0	na	0.19	na

* There was significant heterogeneity in all pooled estimates therefore these should be interpreted with caution

**Figure 3:**
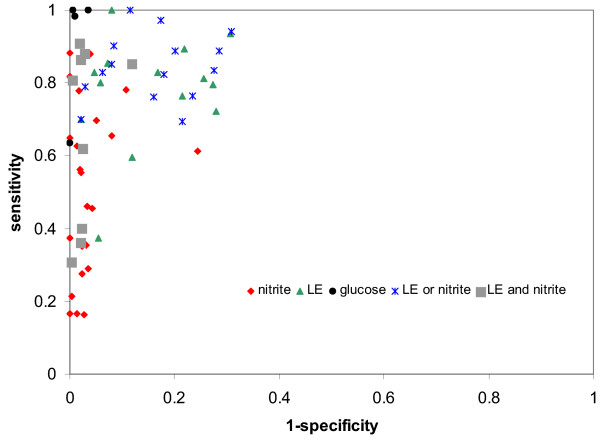
Sensitivity and specificity plotted in ROC space for different dipstick tests.

Figure 3 shows the estimates of sensitivity and 1-specificity plotted in ROC space for glucose, and dipstick tests for nitrite and LE, alone and in combination. This graph suggests that glucose is considerably better than the other tests, both for ruling in and ruling out disease, this is supported by the pooled likelihood ratios. However, the confidence intervals around the pooled likelihood ratios are very large, especially for the negative likelihood ratios (ruling out disease), suggesting considerable uncertainty in these estimates. It should also be noted that very few studies of glucose tests were available and that they were all conducted over 30 years ago and the test used ("Uriglox") [58] is no longer commercially available.

Nitrite alone has a relatively high pooled positive likelihood ratio (15.9, 95% CI: 10.7, 23.7) and so may be useful for ruling in disease. However, it has a relatively poor negative likelihood ratio (0.51, 95% CI: 0.43, 0.60) suggesting that it may not be a useful test for ruling out disease. LE alone appears to be a relatively poor test both for ruling in (pooled LR+ = 5.5, 95% CI: 4.1, 7.3) and ruling out disease (pooled LR- = 0.26, 95% CI: 0.18, 0.36). A strategy which combines the results of LE and nitrite testing appears to offer the best performance both for ruling in and ruling out disease. A dipstick test positive for both nitrite and LE has the highest positive likelihood ratio (28.2, 95% CI: 17.3, 46.0) suggesting that this test combination may be used to rule in disease. A dipstick test negative for both LE and nitrite has the best negative likelihood ratio (0.20, 95% CI: 0.16, 0.26) suggesting that this test combination may be used to rule out disease. A dipstick test positive for either LE or nitrite and negative for the other is less informative for the diagnosis of UTI. Such a test result could be seen as an "indeterminate" test result requiring further investigation.

It is difficult to draw conclusions about the overall accuracy of dipstick tests given the heterogeneity between studies in some areas, and the lack of data in others. There was insufficient information to make any judgement regarding the overall diagnostic accuracy of dipstick tests for protein, blood, or for combinations of three different dipstick tests (e.g. combination of LE, nitrite and blood).

A regression analysis found that only clinical review bias showed an association with the diagnostic accuracy of nitrite dipstick (the DOR was 3.1 (95% CI: 0.97, 9.95) times higher in studies that avoided clinical review bias, i.e. in those studies that reported that the same clinical information was available to those interpreting the test results as would be available in practice). A higher DOR indicates higher overall accuracy. None of the items investigated, including age, showed a significant association with the DOR in the regression analysis for dipstick for LE, or for dipstick for LE or nitrite positive. Regression analysis was not carried out to investigate heterogeneity for other tests, as insufficient data were available.

### Microscopy

A total of 39 studies reporting 101 data sets evaluated microscopy for diagnosing UTI [15-18,26,28-33,35,36,38,42,52,59-61,64,67-84]. Microscopy was used to determine the presence of pyuria or bacteriuria, or combinations of the two. Table 3 summarises the results of these studies.

**Table 3:** Summary of results for studies of microscopy

**Microscopy positive for:**	**Number of studies**	**Range in LR+**	Pooled LR+ (95 % CI)*	**Range in LR-**	Pooled LR- (95 % CI)*
Pyuria	28	1.3 – 27.7	5.9 (4.1, 8.5)	0.04 – 0.68	0.27 (0.20, 0.37)
Bacteriuria	22	1.6 – 304.8	14.7 (8.6, 24.9)	0.01 – 0.48	0.19 (0.14, 0.24)
Pyuria or bacteriuria	8	1.5 – 5.9	4.2 (2.3, 7.6)	0.02 – 0.27	0.11 (0.05, 0.23)
Pyuria and bacteriuria	8	2.7 – 281.0	37.0 (11.0, 125.9)	0.07 – 0.56	0.21 (0.13, 0.36)

* There was significant heterogeneity in all pooled estimates therefore these should be interpreted with caution

**Figure 4:**
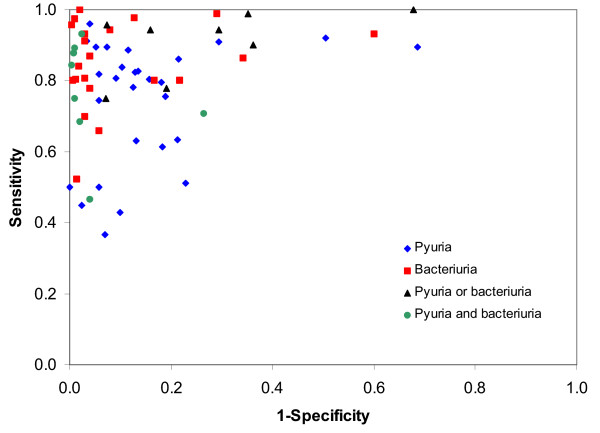
Sensitivity and specificity plotted in ROC space for different microscopy evaluations.

Figure 4 shows the estimates of sensitivity and 1-specificity plotted in ROC space for all studies. This graph suggests that bacteriuria is considerably better than pyuria both for ruling out and ruling in disease. The diagnostic performance of bacteriuria may be improved when combined with pyuria. The pooled positive likelihood ratios are highest for pyuria and bacteriuria combined (37.0, 95% CI: 11.0, 125.9), where a positive result was defined as both tests positive, supporting the suggestion that the combination of a positive result for both of these tests may be useful for ruling in disease. Conversely, the lowest negative likelihood ratio resulted from the combination of pyuria and bacteriuria (0.11, 95% CI: 0.14, 0.24), where a negative result was defined as both tests negative, and this may be useful for ruling out disease. However, the confidence intervals around the pooled estimates are large, and in combination with the observed heterogeneity suggest considerable uncertainty in these estimates.

Regression analysis showed that centrifugation of the sample, reporting of selection criteria, reporting of details of reference standard execution, and reporting of uninterpretable results showed a significant association with the DOR in the studies of microscopy for pyuria. All of these items, with the exception of centrifugation, relate to the quality of reporting. The DOR was 6.25 (95% CI: 3.44, 11.11) times greater in samples that were not centrifuged; 3.19 (95% CI: 1.76, 5.79) times higher in studies that adequately reported selection criteria; 6.6 (95% CI: 2.43, 17.94) times higher in studies that reported sufficient details of reference standard execution; and 2.99 (95% CI: 1.50, 5.94) times higher in studies that reported on uninterpretable results. The association for centrifugation is not what we anticipated, as we would expect centrifugation of the sample to lead to improved test accuracy.

In the analysis of microscopy for bacteriuria, Gram stain, incorporation bias and reporting of selection criteria showed a significant association with the DOR. The DOR was 5.96 (95% CI: 2.99, 11.89) times greater in samples that were Gram stained; 50.0 (95% CI: 6.67, 1000) times greater in studies in which incorporation bias was not present (i.e. studies in which the index test did not form part of the reference standard); and 2.46 (95% CI: 1.26, 8.41) times greater in studies that reported selection criteria. We would expect Gram staining to increase test performance as found in the analysis. However, we would expect the absence of incorporation bias to decrease test performance. The observed association may be explained by the fact that, for the purposes of the regression analysis, studies scoring "unclear" for a quality item were grouped with those scoring "no". For this analysis only one study scored "no" (i.e. incorporation bias was present) and the other studies grouped with this scored as "unclear". The association may therefore reflect quality of reporting, as does the association with reporting of selection criteria.

### Combinations of tests from different categories

Nine studies including a total of 14 data sets examined the accuracy of different combinations of microscopy and dipstick tests for the diagnosis of UTI [30,32,35,36,42,52,67,79,85]. Given the results of individual tests, the test combination that appears to be potentially the most interesting is dipstick for LE and nitrite, and microscopy for pyuria and bacteriuria. Five studies investigated different permutations of these tests. [32,35,42,67,79] Three studies evaluated the accuracy of a positive result in one of these four tests (i.e. dipstick positive for LE or nitrite or microscopy positive for pyuria or bacteriuria) [32,35,67]. The results varied considerably between studies with positive likelihood ratios ranging from 0.8 to 35.9, and negative likelihood ratios ranging from 0.01 to 5.38. It is therefore not possible to draw overall conclusions from these studies. One study examined the combination of a positive result for all four tests [35]. This study reported a very high positive likelihood ratio (35.9) i.e. the combination was found to be very good for ruling in disease, but the negative likelihood ratio was less good at 0.28. These results might be expected given the results from the studies that examined combinations of dipstick tests, or combinations of microscopy tests.

The other test combinations evaluated by these studies differed widely, and none were repeated between studies. Test combinations investigated included LE and nitrite dipstick test combined with microscopy for bacteriruria[42] or pyuria [42,52,85], dipstick for LE, nitrite and blood combined with microscopy for pyuria,[36] and dipstick for nitrite combined with microscopy for pyuria[30].

As most test combinations were only evaluated by one study and the definition of a positive test varied for the tests investigated by more than one study, it was not possible to draw conclusions regarding the diagnostic accuracy of these test combinations.

### Comparison of different tests

Comparison of the pooled likelihood ratios suggests that the microscopy combinations may be more accurate than the dipstick combinations. Only one study evaluated both dipstick positive for nitrite and LE and microscopy positive for bacteriuria and pyuria [59]. This study found that the dipstick combination was best for ruling in disease (LR+ was 18.9 for the dipstick combination compared to 11.6 for the microscopy combination). Five studies examined dipstick negative for nitrite and LE, and microscopy negative for pyuria and bacteriuria [32,35,40,42,59]. All but one found that microscopy was better for ruling out disease than dipstick.

### What do these results mean?

**Figure 5:**
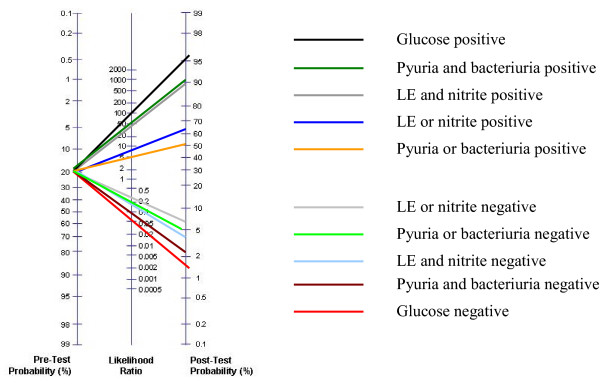
Likelihood ratio nomogram for dipstick tests.

If we take an estimate for the prevalence of UTI in children presenting to their GP with symptoms of possible UTI (the pre-test probability of disease), i.e. children in whom tests to diagnose UTI are likely to be used, likelihood ratios can be used to calculate the post-test probability of UTI. We were unable to find reliable estimates of the pre-test probability of UTI in the literature, and therefore used the results from the included studies to provide an estimate. Only studies that included an appropriate patient spectrum were included in this analysis. UTI prevalence varied greatly between studies (3–73%). As the distribution was highly skewed we used the median prevalence, which was 20%. Figure 5 shows how the probability of UTI changes after testing. In a typical primary care setting in which the pre-test probability of disease is estimated to be around 20%, a negative likelihood ratio of 0.20 translates to a post-test probability of UTI of about 4%. In other words, children who receive a dipstick test negative for both nitrite and LE have a 4% probability of having a UTI.

## Discussion

An accurate and prompt diagnosis is important to inform patient management decisions in young children with suspected UTI. The first step in the diagnostic process is to identify children presenting to the GPs surgery who may have a UTI. This will inevitably involve a clinical assessment. It is very difficult, if not impossible, to capture all the signs and symptoms that a GP might use to develop a clinical suspicion of UTI and decide to test a child for UTI. Further research to accurately define from which children urine samples should be taken to test for UTI may be useful.

Following clinical examination, the next step is to collect a suitable urine sample to test for the presence of infection. Different methods of urine sampling may be differently susceptible to contamination and hence to false positive results. The issue of appropriate urine sampling techniques is of particular concern in young children, where the collection of a sterile, mid-stream sample can be problematic. Suprapubic aspiration has been regarded as the reference standard collection method. This procedure is invasive and may require the use of ultrasound guidance to ensure that the needle is inserted into the bladder. The identification of an alternative sampling method with acceptable diagnostic performance, which can readily be applied in the GP's surgery, and which is more acceptable to children and parents, is therefore desirable. The studies on urine sampling showed reasonably good agreement between clean voided urine (CVU) and suprapubic aspiration (SPA) samples, suggesting that this is an appropriate routine method of urine collection. CVU samples are difficult to collect in young children who are not potty trained. A number of alternative collection methods have been developed, including bag, pad and nappy specimens. There is currently insufficient data available to determine whether bag or nappy/pad specimens may be used as substitutes for SPA Further work is needed in this area.

The main types of urine testing evaluated for the diagnosis of UTI were dipstick and microscopy. Culture is generally considered to be the reference standard for UTI diagnosis. The logistics of urine culture represent a significant drawback; culture takes approximately 48 hours to give a result, is generally performed in the laboratory and is more expensive than other methods. For this reason alternative, more rapid tests are needed to guide the prompt initiation of treatment. Dipsticks have the advantage of providing an immediate result, and of being both cheap and easy to perform and interpret. The studies of dipstick tests showed considerable heterogeneity and so the results should be interpreted with caution. The results suggest that a dipstick test that is positive for both LE and nitrite is good for ruling in disease whilst one that is negative for both LE and nitrite is good for ruling out disease.

An additional dipstick test that provided interesting results was the estimation of urinary glucose, where a negative urinary glucose is regarded as a positive test for UTI. Only four studies of this test were identified, and all were conducted more than 30 years ago. All studies reported excellent specificity for this test. Sensitivity was also very high in three of the studies but was lower, at 64% in the fourth. This last study was conducted in children aged less than one year, suggesting that the test maybe less useful in very young children. This difference in performance of the test with patient age may be explained by its apparent dependence on an overnight, fasting sample; such a sample would be impossible to obtain in children who are not toilet trained. However, given the limited results reported, this test appears to be potentially useful for the diagnosis of UTI in toilet trained children. Further studies are needed.

Although, in practice, microscopy and culture are generally requested in combination, microscopy has the advantage of being quicker to provide a result. It may be that microscopy has some potential as a test that could be performed in the GP surgery. However, it remains more expensive than a dipstick test and requires some degree of expertise to perform. The studies of microscopy showed considerable heterogeneity, in terms of results, cut-off points, types of urine samples and population. A urine sample that was positive for both pyuria and bacteriuria on microscopy was found to be very good for ruling in disease. Similarly, a urine sample that was negative for both pyuria and bacteriuria on microscopy was found to be very good for ruling out disease.

The possibility of publication bias remains a potential problem in this review. It is possible, and indeed likely, that studies reporting higher estimates of test performance are more often published, but the extent to which this occurs is unclear. There is evidence that publication bias is a particular problem for studies of small sample size, although these data are general and does not come from the diagnostic literature[86,87]. We restricted this review to studies that included at least 20 children, meaning that this type of publication bias is less likely to be a problem. We are unaware of any articles on publication bias in diagnostic tests or on methods to formally assess publication bias in a diagnostic systematic review.

We chose likelihood ratios as the primary effect measure as these are the measure that physicians find easiest to interpret [88]. We used pooled likelihood ratios and estimates of the pre-test probability of disease to calculate estimates of the post-test probability of disease. These measures provide a simple illustration of how the results of a test change the probability of disease and help the reader to determine how useful a test is likely to be in practice. The main limitation of this approach was the considerable heterogeneity in pooled likelihood ratios; it is debatable whether it is appropriate to pool these estimates. It is important that pooled estimates are interpreted with caution and that the heterogeneity between studies is considered when interpreting these results. A further problem with this analysis is that positive and negative likelihood ratios were pooled individually. These measures are likely to be correlated within an individual study and ignoring this correlation may be problematic[89].

We conducted a regression analysis to investigate possible explanations for the observed heterogeneity. This analysis was carried out according to standard methods for pooling studies of diagnostic accuracy using the summary ROC approach[14]. Using the DOR for further investigation of heterogeneity means that we can only assess whether the factors investigated are associated with the DOR and not with sensitivity and specificity, or with positive and negative likelihood ratios. Often factors that lead to an increase in sensitivity will lead to a decrease in specificity and vice versa, possibly with no effect on the DOR. A further limitation of this analysis was that we could only investigate the effect of variables at the study level. One factor that may impact on the accuracy of the diagnostic tests investigated is patient age. However, as the majority of studies investigated included children aged 0–16 or 18 years and did not report results separately for younger age groups it was not possible to carry out appropriate sub-group analyses to investigate the effects of age on estimates of test accuracy. This is an area where further investigation is required.

## Conclusion

Based on the results of this review dipstick negative for LE and nitrite, or microscopic analysis negative for pyuria and bacteriuria of a CVU, bag, or nappy/pad specimen may reasonably be used to rule out UTI. These patients can then be excluded from further investigation, without the need for confirmatory culture. Similarly, combinations of positive tests could be used to rule in UTI, and trigger further investigation. In the latter case, however, confirmation by culture may be preferred prior to the initiation of further, possibly invasive, investigations. Additional information on antibiotic sensitivities, which can be provided by culture, may also be a significant consideration. If combinations of rapid tests were routinely used to rule in and/or rule out disease, as described, then a cost saving in the number of cultures ordered would be expected. In addition it is likely that the number of children without disease exposed to inappropriate antibiotic therapy, whilst awaiting culture results, would be reduced. This may have implications for antibiotic resistance at a population level.

The quality assessment highlighted several areas that could be improved upon in future diagnostic accuracy studies, in particular in relation to reporting. Future studies should follow the STARD guidelines for reporting of diagnostic accuracy studies [90].

The review also highlighted the following specific areas requiring further research for the diagnosis of UTI:

• urine sampling methods in younger children

• accuracy of the glucose test, and its practical applicability

• handling of indeterminate nitrite and LE dipstick test results

• accuracy of microscopy in combination with a dipstick test

## Abbreviations

CI Confidence interval

CVU Clean voided urine

DOR Diagnostic odds ratio

ELISA Enzyme linked immunosorbant assay

LE Leukocyte esterase

LR Likelihood ratio

QUADAS Quality assessment of diagnostic accuracy studies

ROC Receiver operating characteristic

SPA Suprapubic aspiration

STARD Standards of reporting of diagnostic accuracy studies

UTI Urinary tract infection

## Competing interests

The author(s) declare that they have no competing interests.

## Authors' contributions

All authors contributed towards the conception and design of the study and the interpretation of the data. They also read and approved the final manuscript. PW and MW participated in data extraction, the analysis of data, and drafted the article.

## Pre-publication history

The pre-publication history for this paper can be accessed here:



## Supplementary Material

Additional File 1 Microsoft Word file.doc containing a table of the results of individual studies included in the review.
Click here for file

Additional File 2 Microsoft Word file.doc containing a table of the results of the quality assessment of included studies.
Click here for file
