# Supplementary material for: Rapid tests and urine sampling techniques for the diagnosis of urinary tract infection (UTI) in children under five years: a systematic review
Source: BMC Pediatr. 2005 Apr 5;5:4. doi: 10.1186/1471-2431-5-4 (PMC1084351; doi:10.1186/1471-2431-5-4)
Supplement: Additional File 1 — Microsoft Word file.doc containing a table of the results of individual studies included in the review. [file 1471-2431-5-4-S1.doc]

## Additional Table 1 Individual study results

| **Study details** | **Test** | **Definition of positive result** | **Reference standard; details; definition of positive result** | | **a** | | | **b** | **c** | | **d** | | **Sens** | **Spec** | **DOR** | **LR+** | | **LR-** | |
| --- | --- | --- | --- | --- | --- | --- | --- | --- | --- | --- | --- | --- | --- | --- | --- | --- | --- | --- | --- |
| **URINE SAMPLING** | | | | | | | | | | | | | | | | | | | |
| **CVU vs SPA: culture** | | | | | | | | | | | | | | | | | | | |
| Aronson (1973)^15^: *Age: <18 months* | | >10^5^ CFU/ml | >=10^2^ CFU/ml | 20 | | | 27 | | 4 | | 35 | | 83.3 | 56.5 | 5.9 | 1.9 | | 0.30 | |
| Aronson (1973)^15^: *Age: 3-12 years* | | >10^5^ CFU/ml | >=10^2^ CFU/ml | 10 | | | 4 | | 4 | | 16 | | 71.4 | 80 | 8.6 | 3.6 | | 0.36 | |
| Hardy (1976)^16^ | | >=10^4^ CFU/ml | >=10^4^ CFU/ml | 3 | | | 1 | | 1 | | 25 | | 75 | 96.2 | 39.7 | 19.5 | | 0.26 | |
| Morton (1982)^17^ | | >10^5^ CFU/ml | Any growth | 5 | | | 0 | | 0 | | 25 | | 100 | 100 | 561 | 47.7 | | 0.08 | |
| Pylkkanen (1979)^18^ | | >=10^4^ CFU/ml | Not clear | 179 | | | 18 | | 20 | | 55 | | 89.9 | 75.3 | 26.3 | 3.6 | | 0.13 | |
| Pylkkanen (1979)^18^ | | >=10^5^ CFU/ml | Not clear | 161 | | | 9 | | 38 | | 64 | | 80.9 | 87.7 | 28.5 | 6.6 | | 0.22 | |
| Ramage (1999)^19^ | | 10^5^ CFU / mL | Any growth | 16 | | | 2 | | 2 | | 38 | | 88.9 | 95 | 101.6 | 17.8 | | 0.12 | |
| **Bag/CVU vs catheter: culture and microscopy** | | | | | | | | | | | | | | | | | | | |
| Braude (1967)^20^: *Aged <5 years* | | Not clear | >=10^4^ CFU/ml and >=10 WBC / mm^3^ | | 17 | | | 4 | 4 | | 26 | | 81.0 | 86.7 | 22.9 | 5.5 | | 0.24 | |
| Braude (1967)^20^: *Whole sample* | | Not clear | >=10^4^ CFU/ml and >=10 WBC / mm^3^ | | 23 | | | 7 | 7 | | 31 | | 76.7 | 81.6 | 13.2 | 3.9 | | 0.30 | |
| **Bag vs SPA: culture** | | | | | | | | | | | | | | | | | | | |
| Benito Fernandez (1996)^21^ | | >10^5^ CFU/ml | >=10^3^ CFU/ml | 13 | | | 4 | | 0 | | 31 | | 100 | 88.6 | 189 | 7.7 | | 0.04 | |
| Hardy (1976)^16^ | | >=10^4^ CFU/ml | >=10^4^ CFU/ml | 2 | | | 2 | | 2 | | 24 | | 50 | 92.3 | 9.8 | 5.4 | | 0.55 | |
| Mendez (2003)^27^ | | >=10^4^ CFU/ml | >=10^4^ CFU/ml. | 27 | | | 72 | | 0 | | 10 | | 100 | 12.2 | 7.97 | 1.1 | | 0.14 | |
| **Pad/nappy vs Bag: culture** | | | | | | | | | | | | | | | | | | | |
| Ahmad (1991)^24^ | | >10^5^ CFU/ml | >=10^5^ CFU/ml | 6 | | | 0 | | 0 | | 39 | | 100 | 100 | 1027 | 74.3 | | 0.07 | |
| Farrell (2002)^22^ | | Bacteria present | Bacteria present | 2 | | | 0 | | 3 | | 15 | | 40 | 100 | 22.1 | 13.3 | | 0.60 | |
| Feasey (1999)^23^ | | >= 10^5^ CFU/ml | >=10^5^ CFU/ml | 2 | | | 0 | | 0 | | 44 | | 100 | 100 | 445 | 75.0 | | 0.17 | |
| **Pad/nappy vs SPA: culture** | | | | | | | | | | | | | | | | | | | |
| Cohen (1997)^25^ | | >10^5^ CFU/ml | >=10^5^ CFU/ml | 5 | | | 2 | | 0 | | 31 | | 100 | 93.9 | 138.6 | 12.5 | | 0.09 | |
| **Early vs late stream catheter sample: culture** | | | | | | | | | | | | | | | | | | | |
| Dayan (2000)^26^ | | >=50000 CFU/ml. | >=50000 CFU/ml. | 6 | | | 4 | | 0 | | 76 | | 100 | 95.0 | 221.0 | 16.7 | | 0.08 | |
| **DIPSTICK TESTS** | | | | | | | | | | | | | | | | | | | |
| **Nitrite vs culture** | | | | | | | | | | | | | | | | | | | |
| Armengol (2001)^29^ | Ames/BM | | >=10^4^ CFU/ml | | 5 | | | 0 | 25 | | 230 | | 16.7 | 100.0 | 99.4 | 82.0 | | 0.83 | |
| Boreland (1986)^46^ | N-Labstix (ames) | | >=10^5^ CFU/ml | | 37 | | | 10 | 30 | | 459 | | 55.2 | 97.9 | 53.8 | 25.9 | | 0.46 | |
|  | N-Labstix (ames); read by Clinitek 200 | |  |  | 8 | | | 2 | 7 | | 147 | | 53.3 | 98.7 | 66.9 | 31.9 | | 0.48 | |
| Bulloch (2000)^32^ | Clinitek (Ames) | | Catheter: >10^4^, CVU: >=10^5^ CFU/ml | | 8 | | | 3 | 21 | | 127 | | 27.6 | 97.7 | 14.4 | 12.0 | | 0.74 | |
| Dayan (2002)^28^ | Automated urine analysis; Super UA | | SPA: >=10^3^, Catheter: >=10^4^CFU/ml | | 7 | | | 4 | 13 | | 169 | | 35.0 | 97.7 | 20.9 | 15.1 | | 0.67 | |
| Demi (1993)^41^ | BM | | Not reported | | 4 | | | 3 | 20 | | 220 | | 16.7 | 98.7 | 13.8 | 12.4 | | 0.84 | |
| Fennell (1977)^49^ | Bac-U-Dip (Warner-Chilcutt) | | >=10^5^ CFU/ml | | 7 | | | 1 | 2 | | 55 | | 77.8 | 98.2 | 111.0 | 43.6 | | 0.23 | |
| Benito Fernandez (2000)^30^ | Multistix (Bayer) | | >50 000 cfu/ml | | 40 | | | 3 | 47 | | 85 | | 46.0 | 96.6 | 20.8 | 13.5 | | 0.56 | |
| Giraldez (1998)^64^ | Not reported | | >=10^5^ CFU/ml | | 22 | | | 1 | 3 | | 24 | | 88.0 | 96.0 | 105.0 | 22.0 | | 0.13 | |
| Holland (1968)^56^ | Stat-Test (Mallinckrodt) | | >=10^5^ CFU/ml | | 13 | | | 0 | 7 | | 106 | | 65.0 | 100.0 | 383.4 | 137.6 | | 0.35 | |
| Kunin (1977)^50^ | Microstix-Nitrite (Ames) | | >=10^5^ CFU/ml | | 18 | | | 0 | 4 | | 55 | | 81.8 | 100.0 | 456.3 | 90.1 | | 0.18 | |
| Labbe (1982)^48^ | N-Uristix (Ames); Home based (mothers) | | >=10^5^ CFU/ml | | 37 | | | 0 | 5 | | 89 | | 88.1 | 100.0 | 1221 | 157.0 | | 0.12 | |
| Liptak (1993)^40^ | Chemstrip 9 (BM) | | >=10^5^ CFU/ml | | 68 | | | 18 | 36 | | 207 | | 65.4 | 92.0 | 21.0 | 8.2 | | 0.38 | |
| Lohr (1993)^42^ | Ames; read with Clinitek | | SPA: >10^3^, catheter: >10^4^ CVU >10^5^ CFU/ml | | 38 | | | 0 | 64 | | 587 | | 37.3 | 100.0 | 701.4 | 439.6 | | 0.63 | |
| Marsik (1986)^47^ | Chemstrip (biodynamics) | | SPA: any bacteria; Catheter: >10^4^, CVU: >10^5^ CFU/ml | | 37 | | | 28 | 16 | | 520 | | 69.8 | 94.9 | 41.5 | 13.7 | | 0.32 | |
| Ordonez (1994)^54^ | Ames (multristx) | | >=10^5^ CFU/ml | | 11 | | | 20 | 7 | | 62 | | 61.1 | 75.6 | 4.7 | 2.5 | | 0.51 | |
| Parmington (1989)^63^ | Not reported | | Not stated | | 43 | | | 27 | 12 | | 223 | | 78.2 | 89.2 | 28.3 | 7.2 | | 0.24 | |
| Sharief (1998)^34^  *Total population* | Multistix (bayer); automated reader | | >=10^5^ CFU/ml | | 6 | | | 10 | 11 | | 298 | | 35.3 | 96.8 | 16.1 | 10.9 | | 0.67 | |
| *infants < 1 yr* |  |  |  |  | 1 | | | 2 | 7 | | 114 | | 12.5 | 98.3 | 9.2 | 7.8 | | 0.85 | |
| Tahirovic (1988)^44^ | Urocomb (BM) | | >=10^5^ CFU/ml | | 16 | | | 1 | 59 | | 230 | | 21.3 | 99.6 | 42.6 | 49.3 | | 0.79 | |
|  | Urocomb (BM); negative samples incubated for 4hr at 37^0^C | |  |  | 55 | | | 20 | 20 | | 211 | | 73.3 | 91.3 | 27.9 | 8.3 | | 0.30 | |
|  | Urocomb (BM); negative samples incubated with NaNO3 for 4hr at 37^0^C | |  |  | 70 | | | 5 | 27 | | 204 | | 72.2 | 97.6 | 95.3 | 27.5 | | 0.29 | |
| Wammanda (2000)^31^ | Multistix (Bayer) | | >=10^5^ CFU/ml | | 13 | | | 5 | 32 | | 135 | | 28.9 | 96.4 | 10.2 | 8.1 | | 0.74 | |
| Weinberg (1991)^60^ | Multistix (Ames) | | >=10^5^ CFU/ml | | 23 | | | 19 | 18 | | 959 | | 56.1 | 98.1 | 62.5 | 28.9 | | 0.45 | |
| Wiggelinkhuizen (1988)^45^ | Combur 9 (BM) | | Not clear | | 96 | | | 9 | 57 | | 800 | | 62.7 | 98.9 | 141.4 | 46.1 | | 0.38 | |
|  | Multistix (Ames) | |  |  | 96 | | | 11 | 57 | | 798 | | 62.7 | 98.6 | 116.5 | 44.1 | | 0.38 | |
| **Nitrite vs culture and microscopy** | | | | | | | | | | | | | | | | | | | |
| Lagos Zuccone (1994)^65^ | Combur-9 (BM) | | >=10^5^ CFU/ml and >10 cells/mm^3^ | | 158 | | | 27 | 190 | | 615 | | 45.4 | 95.8 | 18.6 | 10.8 | | 0.57 | |
| Lejeune (1991)^43^ | Multistick (Ames); read by Clinitek | | various WBC/ml and >10^5^ CFU/ml | | 6 | | | 5 | 31 | | 201 | | 16.2 | 97.6 | 7.6 | 6.7 | | 0.86 | |
| **LE vs culture** | | | | | | | | | | | | | | | | | | | |
| Armengol (2001)^29^ | Ames/BM | | >=10^4^ CFU/ml | | 21 | | | 5 | 9 | | 225 | | 70.0 | 97.8 | 92.8 | 32.2 | | 0.31 | |
| Bulloch (2000)^32^ | Clinitek (Ames) | | Catheter: >10^4^, CVU: >=10^5^ CFU/ml | | 24 | | | 6 | 5 | | 124 | | 82.8 | 95.4 | 85.3 | 17.9 | | 0.18 | |
| Dayan (2002)^28^ | Automated urine analysis; Super UA | | SPA: >=10^3^, Catheter: >=10^4^CFU/ml | | 16 | | | 10 | 4 | | 163 | | 80.0 | 94.2 | 57.1 | 13.8 | | 0.21 | |
| Demi (1993)^41^ | BM | | Not reported | | 9 | | | 12 | 15 | | 211 | | 37.5 | 94.6 | 10.4 | 7.0 | | 0.66 | |
| Giraldez (1998)^64^ | Not reported | | >=10^5^ CFU/ml | | 25 | | | 2 | 0 | | 23 | | 100.0 | 92.0 | 479.4 | 12.5 | | 0.02 | |
| Liptak (1993)^40^ | Chemstrip 9 (BM) | | >=10^5^ CFU/ml | | 62 | | | 27 | 42 | | 198 | | 59.6 | 88.0 | 10.6 | 5.0 | | 0.46 | |
| Lohr (1993)^42^ | Ames; read with Clinitek | | SPA: >10^3^, catheter: >10^4^ CVU >10^5^ CFU/ml | | 81 | | | 160 | 21 | | 427 | | 79.4 | 72.7 | 10.1 | 2.9 | | 0.28 | |
| Marsik (1986)^47^ | Chemstrip (biodynamics) | | SPA: any bacteria; Catheter: >10^4^, CVU: >10^5^ CFU/ml | | 43 | | | 140 | 10 | | 408 | | 81.1 | 74.5 | 12.0 | 3.2 | | 0.25 | |
| Villanustre Ordonez (1994)^54^ | Ames (multristx) | | >=10^5^ CFU/ml | | 13 | | | 23 | 5 | | 59 | | 72.2 | 72.0 | 6.2 | 2.6 | | 0.39 | |
| Sharief (1998)^34^  *Total population* | Multistix (bayer); automated reader | | >=10^5^ CFU/ml | | 13 | | | 66 | 4 | | 242 | | 76.5 | 78.6 | 10.9 | 3.6 | | 0.30 | |
| *infants < 1 yr* |  |  |  |  | 6 | | | 30 | 2 | | 86 | | 75.0 | 74.1 | 7.4 | 2.8 | | 0.38 | |
| Weinberg (1991)^60^ | Multistix (Ames) | | >=10^5^ CFU/ml | | 35 | | | 71 | 6 | | 907 | | 85.4 | 92.7 | 69.3 | 11.8 | | 0.16 | |
| Wiggelinkhuizen (1988)^45^ | Multistix (Ames) | | Not clear | | 143 | | | 248 | 10 | | 561 | | 93.5 | 69.3 | 30.9 | 3.0 | | 0.09 | |
|  | Combur 9 (BM) | |  |  | 144 | | | 252 | 9 | | 557 | | 94.1 | 68.9 | 33.6 | 3.0 | | 0.09 | |
| **LE vs culture and microscopy** | | | | | | | | | | | | | | | | | | | |
| Lagos Zuccone (1994)^65^ | Combur-9 (BM) | | >=10^5^ CFU/ml and >10 cells/mm^3^ | | 289 | | | 107 | 59 | | 535 | | 83.0 | 83.3 | 24.2 | 5.0 | | 0.20 | |
| Lejeune (1991)^43^ | Multistick (Ames); read by Clinitek | | various WBC/ml and >10^5^ CFU/ml | | 33 | | | 45 | 4 | | 161 | | 89.2 | 78.2 | 26.4 | 4.1 | | 0.14 | |
| **Protein vs culture** | | | | | | | | | | | | | | | | | | | |
| Boreland (1986)^46^ | N-Labstix (ames) | | >=10^5^ CFU/ml | | 27 | | | 109 | 40 | | 360 | | 40.3 | 76.8 | 2.2 | 1.7 | | 0.78 | |
|  | N-Labstix (ames); read by Clinitek 200 | |  |  | 8 | | | 24 | 7 | | 125 | | 53.3 | 83.9 | 5.8 | 3.3 | | 0.56 | |
| **Protein vs culture and microscopy** | | | | | | | | | | | | | | | | | | | |
| Lejeune (1991)^43^ | Multistick (Ames); read by Clinitek | | various WBC/ml and >10^5^ CFU/ml | | 3 | | | 10 | 34 | | 196 | | 8.1 | 95.1 | 1.9 | 1.8 | | 0.96 | |
| **Glucose vs culture** | | | | | | | | | | | | | | | | | | | |
| Dosa (1973)^55^  Group A | Uriglox | | >=10^5^ CFU/ml | | 2 | | | 1 | 63 | | 356 | | 3.1 | 99.7 | 9.4 | 9.0 | | 0.97 | |
| Group B |  |  |  |  | 7 | | | 0 | 4 | | 25 | | 63.6 | 100.0 | 85.0 | 32.5 | | 0.36 | |
| Kohler (1970)^57^ | Uriglox | | >=10^5^ CFU/ml | | 22 | | | 12 | 0 | | 1982 | | 100.0 | 99.4 | 7137 | 166.2 | | 0.02 | |
| Schersten (1968)^58^ | Not reported | | >=10^5^ CFU/ml | | 6 | | | 19 | 0 | | 510 | | 100.0 | 96.4 | 340.3 | 27.8 | | 0.07 | |
| Todd (1974)^51^ | Uriglox | | >=10^3^ catheter and >=10^5^ CFU/ml CVU | | 55 | | | 5 | 1 | | 574 | | 98.2 | 99.1 | 3865 | 113.7 | | 0.02 | |
| **Blood vs culture** | | | | | | | | | | | | | | | | | | | |
| Boreland (1986)^46^ | N-Labstix (ames) | | >=10^5^ CFU/ml | | 17 | | | 52 | 50 | | 417 | | 25.4 | 88.9 | 2.8 | 2.3 | | 0.84 | |
|  | N-Labstix (ames); read by Clinitek 200 | |  |  | 8 | | | 23 | 7 | | 126 | | 53.3 | 84.6 | 6.1 | 3.4 | | 0.56 | |
| **Nitrite or LE positive vs culture** | | | | | | | | | | | | | | | | | | | |
| Anad (2001)^52^ | Nephu-Test + Leuco (BM) | | Culture; SPA: any; other >10 CFU/ml | | 34 | | | 95 | 15 | | 346 | | 69.4 | 78.5 | 8.1 | 3.2 | | 0.39 | |
| Armengol (2001)^29^ | Ames/BM | | >=10^4^ CFU/ml | | 21 | | | 5 | 9 | | 225 | | 70.0 | 97.8 | 92.8 | 32.2 | | 0.31 | |
| Bulloch (2000)^32^ | Clinitek (Ames) | | Catheter: >10^4^, CVU: >=10^5^ CFU/ml | | 24 | | | 8 | 5 | | 122 | | 82.8 | 93.8 | 64.2 | 13.4 | | 0.18 | |
| Dayan (2002)^28^ | Automated urine analysis; Super UA | | SPA: >=10^3^, Catheter: >=10^4^CFU/ml | | 17 | | | 14 | 3 | | 159 | | 85.0 | 91.9 | 55.0 | 10.5 | | 0.16 | |
| Liptak (1993)^40^ | Chemstrip 9 (BM) | | >=10^5^ CFU/ml | | 79 | | | 36 | 25 | | 189 | | 76.0 | 84.0 | 16.2 | 4.7 | | 0.29 | |
| Lohr (1993)^42^ | Ames; read with Clinitek | | SPA: >10^3^, catheter: >10^4^ CVU >10^5^ CFU/ml | | 85 | | | 162 | 17 | | 425 | | 83.3 | 72.4 | 12.8 | 3.0 | | 0.23 | |
| Marsik (1986)^47^ | Chemstrip (biodynamics) | | SPA: any bacteria; Catheter: >10^4^, CVU: >10^5^ CFU/ml | | 47 | | | 156 | 6 | | 392 | | 88.7 | 71.5 | 18.3 | 3.1 | | 0.16 | |
| Sharief (1998)^34^  *infants < 1 yr* | Multistix (bayer); automated reader | | >=10^5^ CFU/ml | | 6 | | | 31 | 2 | | 85 | | 75.0 | 73.3 | 7.1 | 2.7 | | 0.38 | |
| *Total population* |  |  |  |  | 13 | | | 72 | 4 | | 236 | | 76.5 | 76.6 | 9.8 | 3.3 | | 0.31 | |
| Shaw (1991)^59^  <2 years | Multistix; at least trace LE | | Catheter: >=10^3^, CVU: >=10^5^ CFU/ml. | | 10 | | | 10 | 4 | | 121 | | 71.4 | 92.4 | 27.0 | 8.8 | | 0.33 | |
| All children | Multistix; at least trace LE | |  |  | 37 | | | 80 | 8 | | 366 | | 82.2 | 82.1 | 20.1 | 4.6 | | 0.22 | |
|  | Multistix; at least small LE | |  |  | 36 | | | 58 | 9 | | 388 | | 80.0 | 87.0 | 25.5 | 6.1 | | 0.24 | |
| Shaw (1998)^35^ | Multistix (Bayer); at least moderate LE | | >=10^4^ CFU/ml | | 69 | | | 33 | 26 | | 3266 | | 72.6 | 99.0 | 255.7 | 71.3 | | 0.28 | |
|  | Multistix (Bayer); at least trace LE | |  |  | 75 | | | 99 | 20 | | 3200 | | 78.9 | 97.0 | 118.5 | 26.3 | | 0.22 | |
| Waisman (1999)^33^ | Multistix (Bayer) | | SPA, catheter: >10^3^, CVU/bag: >10^5^ CFU/ml | | 34 | | | 15 | 1 | | 71 | | 97.1 | 82.6 | 106.1 | 5.6 | | 0.03 | |
| Weinberg (1991)^60^ | Multistix (Ames) | | >=10^5^ CFU/ml | | 37 | | | 82 | 4 | | 896 | | 90.2 | 91.6 | 90.6 | 10.6 | | 0.11 | |
| Wiggelinkhuizen (1988)^45^ | Multistix (Ames) | | Culture; Not clear | | 144 | | | 249 | 9 | | 560 | | 94.1 | 69.2 | 34.2 | 3.1 | | 0.08 | |
|  | Combur 9 (BM) | |  |  | 146 | | | 253 | 7 | | 556 | | 95.4 | 68.7 | 42.9 | 3.0 | | 0.07 | |
| **Nitrite or LE positive vs culture and microscopy** | | | | | | | | | | | | | | | | | | | |
| Lagos Zuccone (1994)^65^ | Combur-9 (BM) | | >=10^5^ CFU/ml and >10 cells/mm^3^ | | 309 | | | 129 | 39 | | 513 | | 88.8 | 79.9 | 31.1 | 4.4 | | 0.14 | |
| Woodward (1993)^39^ | Multistix (Bayer) | | >=10^5^ CFU/ml and >=20 WBC/mm^3^ | | 12 | | | 14 | 0 | | 107 | | 100.0 | 88.4 | 185.3 | 8.6 | | 0.04 | |
| **Nitrite and LE positive vs culture** | | | | | | | | | | | | | | | | | | | |
| Dayan (2002)^28^ | Automated urine analysis; Super UA | | SPA: >=10^3^, Catheter: >=10^4^CFU/ml | | 6 | | | 0 | 14 | | 173 | | 30.0 | 100.0 | 155.6 | 107.7 | | 0.69 | |
| Giraldez (1998)^64^ | not reported | | >=10^5^ CFU/ml | | 22 | | | 0 | 3 | | 25 | | 88.0 | 100.0 | 327.9 | 45.0 | | 0.14 | |
| Marret (1995)^62^ | Combur-9 (BM) | | Not clear | | 20 | | | 5 | 3 | | 41 | | 87.0 | 89.1 | 44.2 | 7.3 | | 0.17 | |
| Marsik (1986)^47^ | Chemstrip (biodynamics) | | SPA: any bacteria; Catheter: >10^4^, CVU: >10^5^ CFU/ml | | 33 | | | 12 | 20 | | 536 | | 62.3 | 97.8 | 70.1 | 27.2 | | 0.39 | |
| Sharief (1998)^34^  *infants < 1 yr* | Multistix (bayer); automated reader | | >=10^5^ CFU/ml | | 1 | | | 1 | 7 | | 115 | | 12.5 | 99.1 | 15.4 | 13.0 | | 0.84 | |
| *Total population* |  |  |  |  | 6 | | | 4 | 11 | | 304 | | 36.1 | 98.5 | 38.3 | 24.8 | | 0.65 | |
| Shaw (1991)^59^  *<2 years* | Multistix | | Catheter: >=10^3^, CVU: >=10^5^ CFU/ml. | | 2 | | | 3 | 12 | | 128 | | 14.3 | 97.7 | 7.3 | 6.3 | | 0.86 | |
| *All children* |  |  |  |  | 18 | | | 9 | 27 | | 437 | | 40.0 | 98.0 | 31.0 | 18.9 | | 0.61 | |
| Wiggelinkhuizen (1988)^45^ | Multistix (Ames) | | Not clear | | 95 | | | 10 | 58 | | 799 | | 62.0 | 98.7 | 124.3 | 47.8 | | 0.38 | |
|  | Combur 9 (BM) | |  |  | 94 | | | 8 | 89 | | 801 | | 61.4 | 99.0 | 149.8 | 58.5 | | 0.39 | |
| **Nitrite and LE positive vs culture and microscopy** | | | | | | | | | | | | | | | | | | | |
| Lejeune (1991)^43^ | Multistick (Ames); read by Clinitek | | various WBC/ml and >10^5^ CFU/ml | | 33 | | | 5 | 4 | | 201 | | 89.2 | 97.6 | 272.7 | 33.2 | | 0.12 | |
| Woodward (1993)^39^ | Multistix (Bayer) | | >=10^5^ CFU/ml and >=20 WBC/mm^3^ | | 10 | | | 0 | 2 | | 121 | | 83.3 | 100.0 | 1021 | 197.1 | | 0.19 | |
| **LE and protein positive vs culture and microscopy** | | | | | | | | | | | | | | | | | | | |
| Lejeune (1991)^43^ | Multistick (Ames); read by Clinitek | | various WBC/ml and >10^5^ CFU/ml | | 33 | | | 10 | 4 | | 196 | | 89.2 | 95.1 | 139.3 | 17.4 | | 0.12 | |
| **Nitrite, blood or protein positive vs culture** | | | | | | | | | | | | | | | | | | | |
| Boreland (1986)^46^ | N-Labstix (ames); read by Clinitek 200 | | Culture; >=10^5 CFU/ml | | 14 | | | 38 | 1 | | 111 | | 93.3 | 74.5 | 28.0 | 3.5 | | 0.13 | |
|  | N-Labstix (ames) | |  |  | 54 | | | 138 | 13 | | 331 | | 80.6 | 70.6 | 9.7 | 2.7 | | 0.28 | |
| **Nitrite, blood or LE positive vs culture** | | | | | | | | | | | | | | | | | | | |
| Rodriguez Cervilla (2001)^53^ | Not stated | | SPA: >=10^2^, catheter: >=10^4^, CVU: >=10^5^ CFU/ml | | 36 | | | 39 | 7 | | 20 | | 83.7 | 33.9 | 2.5 | 1.3 | | 0.50 | |
| **Nitrite, blood and LE positive vs culture** | | | | | | | | | | | | | | | | | | | |
| Craver (1997)^36^ | Multistix (Ames): at least trace blood | | Catheter: >10^3^, CVU/bag: >50 000 CFU/ml | | 22 | | | 80 | 2 | | 132 | | 91.7 | 62.3 | 14.8 | 2.4 | | 0.16 | |
|  | Multistix (Ames): at least blood 1+ | |  |  | 21 | | | 52 | 3 | | 160 | | 87.5 | 75.5 | 18.8 | 3.5 | | 0.19 | |
| **Nitrite, LE and protein positive vs culture** | | | | | | | | | | | | | | | | | | | |
| Wiggelinkhuizen (1988)^45^ | Multistix (Ames) | | Not clear | | 80 | | | 5 | 3 | | 391 | | 96.4 | 98.7 | 1637 | 69.2 | | 0.04 | |
|  | Combur 9 (BM) | |  |  | 68 | | | 4 | 3 | | 437 | | 95.8 | 99.1 | 1903 | 93.4 | | 0.05 | |
| **Nitrite, LE and protein positive vs culture and microscopy** | | | | | | | | | | | | | | | | | | | |
| Lejeune (1991)^43^ | Multistick (Ames); read by Clinitek | | various WBC/ml and >10^5^ CFU/ml | | 33 | | | 58 | 4 | | 148 | | 89.2 | 71.8 | 18.9 | 3.1 | | 0.17 | |
| **Nitrite, LE or protein positive vs culture** | | | | | | | | | | | | | | | | | | | |
| Wiggelinkhuizen (1988)^45^ | Multistix (Ames) | | Not clear | 150 | | 418 | | | 3 | 391 | | 98.0 | | 48.3 | 40.2 | 1.9 | | 0.05 | |
|  | Combur 9 (BM) | |  | 150 | | 372 | | | 3 | 437 | | 98.0 | | 54.0 | 50.5 | 2.1 | | 0.04 | |
| **Nitrite, LE, blood or protein positive vs culture** | | | | | | | | | | | | | | | | | | | |
| Doley (2003)^66^ | Clinitek 50 (Bayer) | | >10^5^ CFU/ml of an isolated organism | | 37 | | | 203 | 3 | | 132 | | 92.5 | 39.4 | 1.5 | 0.19 | | 8.02 | |
| **MICROSCOPY** | | | | | | | | | | | | | | | | | | | |
| **Pyuria vs Culture** | | | | | | | | | | | | | | | | | | | |
| Anad (2001)^52^ | Uncentrifuged | Any WBC/hpf | SPA: any, other >10 CFU/ml | | 37 | | 83 | | 12 | 358 | | 75.5 | | 81.2 | 12.9 | | 4.0 | | 0.30 |
| Aronson (1973)^15^  *3-12 yrs* | SPA | >10 WBC/mm^3^ | SPA: >=10^2^ CFU/ml | | 13 | | 6 | | 1 | 14 | | 92.9 | | 70.0 | 20.1 | | 2.9 | | 0.14 |
|  | CVU | >250 WBC/mm^3^ |  |  | 13 | | 9 | | 1 | 11 | | 92.9 | | 55.0 | 10.9 | | 2.0 | | 0.18 |
| *<18 mths* | CVU | >250 WBC/mm^3^ |  |  | 20 | | 0 | | 4 | 62 | | 83.3 | | 100.0 | 569.4 | | 103.3 | | 0.18 |
|  | SPA | >10 WBC/mm^3^ |  |  | 21 | | 0 | | 3 | 62 | | 87.5 | | 100.0 | 767.9 | | 108.4 | | 0.14 |
| Arslan (2002)^67^ | Centrifuged | > 5 WBC | >=10^5^ CFU/ml | | 30 | | 3 | | 40 | 27 | | 42.9 | | 90.0 | 5.9 | | 4.3 | | 0.63 |
| Bulloch (2000)^32^ | Centrifuged | >=5WBC/hpf | Catheter: >10^4^ , CVU: >=10^5^ CFU/ml | | 25 | | 28 | | 4 | 102 | | 86.2 | | 78.5 | 20.4 | | 4.0 | | 0.18 |
| Rodriguez Caballero (2001)^68^ | Centrifuged | Any WBC | >=10^5^ CFU/ml | | 80 | | 193 | | 7 | 190 | | 92.0 | | 49.6 | 10.6 | | 1.8 | | 0.16 |
|  |  | >18 WBC |  |  | 72 | | 62 | | 15 | 321 | | 82.8 | | 83.8 | 24.1 | | 5.1 | | 0.21 |
| Dayan (2000)^26^ | Early sample;  Centrifuged | >=5 WBC/hpf | Catheter late stream sample: >=50000 CFU/ml. | | 4 | | 20 | | 2 | 59 | | 66.7 | | 74.7 | 5.2 | | 2.5 | | 0.48 |
|  |  | >=10 WBC/hpf |  |  | 3 | | 5 | | 3 | 74 | | 50.0 | | 93.7 | 13.5 | | 7.3 | | 0.54 |
|  | Late sample;  Centrifuged | >=10 WBC/hpf |  |  | 3 | | 4 | | 3 | 75 | | 50.0 | | 94.9 | 16.8 | | 8.9 | | 0.53 |
|  |  | >=5 WBC/hpf |  |  | 5 | | 10 | | 1 | 69 | | 83.3 | | 87.3 | 24.3 | | 6.0 | | 0.25 |
| Benito Fernandez (2000)^30^ | Not stated | Not stated | >50 000 CFU/ml | | 79 | | 26 | | 8 | 62 | | 90.8 | | 70.5 | 22.1 | | 3.1 | | 0.13 |
| Giraldez (1998)^64^ | Centrifuged | >=10 WBC/hpf | >=10^5^ CFU/ml | | 24 | | 1 | | 1 | 24 | | 96.0 | | 96.0 | 266.8 | | 24.0 | | 0.04 |
| Hardy (1976)^16^ | CVU | >10 WBC/mm^3^ | SPA: >=10^4^ CFU/ml | | 2 | | 1 | | 2 | 25 | | 50.0 | | 96.2 | 17.0 | | 9.0 | | 0.53 |
|  | Bag |  |  |  | 1 | | 1 | | 3 | 25 | | 25.0 | | 96.2 | 7.3 | | 5.4 | | 0.74 |
|  | SPA |  |  |  | 2 | | 0 | | 2 | 26 | | 50.0 | | 100.0 | 53.0 | | 27.0 | | 0.50 |
| Hiraoka (1995)^69^ | Not stated | >=10 WBC/mm^3^ | Catheter: >10^3^, CVU: >10^5^ CFU/ml | | 19 | | 9 | | 4 | 57 | | 82.6 | | 86.4 | 26.2 | | 6.1 | | 0.20 |
| Hoberman (1996)^70^ | Uncentrifuged | >=10 WBC/mm^3^ | >=50 000 CFU/ml | | 190 | | 206 | | 22 | 3835 | | 89.6 | | 94.9 | 157.3 | | 17.6 | | 0.11 |
|  |  | > 10 WBC/mm^3^ | >=50000 CFU/ml | | 93 | | 67 | | 9 | 1969 | | 91.2 | | 96.7 | 287.2 | | 27.7 | | 0.09 |
| Lin (2000)^72^ | Uncentrifuged | >=10 WBC/μl | >=10^2^ CFU/ml | | 18 | | 8 | | 4 | 132 | | 81.8 | | 94.3 | 64.1 | | 14.3 | | 0.19 |
|  | Centrifuged | >=5 WBC/hpf |  |  | 13 | | 10 | | 9 | 130 | | 59.1 | | 92.9 | 17.7 | | 7.9 | | 0.45 |
| Lin (2000)^73^ | Uncentrifuged | >=10 WBC/μl | >=10^3^ CFU/ml | | 31 | | 20 | | 6 | 173 | | 83.8 | | 89.6 | 41.0 | | 8.1 | | 0.18 |
|  | Centrifuged | >=5 WBC/hpf |  |  | 24 | | 23 | | 13 | 170 | | 64.9 | | 88.1 | 13.2 | | 5.3 | | 0.40 |
| Lohr (1993)^42^ | Centrifuged | >5 WBC/hpf | SPA: >10^3^, catheter: >10^4^, CVU >10^5^ CFU/ml | | 82 | | 92 | | 20 | 495 | | 80.4 | | 84.3 | 21.6 | | 5.1 | | 0.23 |
| Matthai (1995)^38^ | Centrifuged | >5 WBC/hpf | >=10^5^ CFU/ml | | 190 | | 50 | | 36 | 100 | | 84.1 | | 66.7 | 10.4 | | 2.5 | | 0.24 |
|  |  | >20 WBC/hpf |  |  | 49 | | 9 | | 77 | 141 | | 38.9 | | 94.0 | 9.5 | | 6.2 | | 0.65 |
|  |  | >10 WBC/hpf |  |  | 180 | | 27 | | 46 | 123 | | 79.6 | | 82.0 | 17.4 | | 4.4 | | 0.25 |
| Morton (1982)^17^ | Uncentrifuged | >10 WBC/mm^3^ | SPA: any, CVU: >10^5^ CFU/ml | | 43 | | 32 | | 25 | 210 | | 63.2 | | 86.8 | 11.0 | | 4.7 | | 0.42 |
| Pryles (1965)^74^ | Uncentrifuged | >=100 WBC/mm^3^ | >=10^5^ CFU/ml | | 10 | | 2 | | 34 | 229 | | 22.7 | | 99.1 | 27.9 | | 21.7 | | 0.78 |
|  |  | >=10 WBC/mm^3^ |  |  | 27 | | 42 | | 17 | 189 | | 61.4 | | 81.8 | 7.0 | | 3.4 | | 0.47 |
|  | Centrifuged | >=5 WBC/mm^3^ |  |  | 19 | | 6 | | 25 | 235 | | 43.2 | | 97.5 | 27.7 | | 16.1 | | 0.58 |
| Pylkkanen (1979)^18^ | CVU | >=11 WBC/mm^3^ | SPA | | 178 | | 50 | | 21 | 23 | | 89.4 | | 31.5 | 3.9 | | 1.3 | | 0.33 |
|  |  | >=200 WBC/mm^3^ |  |  | 117 | | 16 | | 82 | 57 | | 58.8 | | 78.1 | 5.0 | | 2.6 | | 0.53 |
| Santos (1982)^75^ | Centrifuged; stained | Not stated | Not stated | | 233 | | 95 | | 403 | 1269 | | 36.6 | | 93.0 | 7.7 | | 5.3 | | 0.68 |
| Saxena (1975)^76^ | Uncentrifuged | >=10 WBC/mm^3^ | Not stated | | 21 | | 4 | | 5 | 40 | | 80.8 | | 90.9 | 35.2 | | 8.9 | | 0.21 |
| Schreiter (1971)^77^  *<1yr Antibiotics* | Not stated | >20 WBC/μl | >=10^5^ CFU/ml | | 5 | | 14 | | 4 | 67 | | 55.6 | | 82.7 | 5.7 | | 3.1 | | 0.55 |
| *<1yr No antibiotics* |  |  |  |  | 51 | | 7 | | 14 | 237 | | 78.5 | | 97.1 | 112.5 | | 25.5 | | 0.23 |
| *1-14yrs Antibiotics* |  |  |  |  | 2 | | 22 | | 5 | 69 | | 28.6 | | 75.8 | 1.4 | | 1.3 | | 0.91 |
| *1-14yrs No antibiotics* |  |  |  |  | 53 | | 6 | | 15 | 438 | | 77.9 | | 98.6 | 232.9 | | 53.1 | | 0.23 |
| Shaw (1998)^35^ | Uncentrifuged | >= 10 WBC/mm^3^ | >=10^4^ CFU/ml | | 47 | | 278 | | 10 | 1858 | | 82.5 | | 87.0 | 30.2 | | 6.3 | | 0.20 |
| Wammanda (2000)^31^ | Centrifuged | >=10 WBC/hpf | >=10^5^ CFU/ml | | 23 | | 32 | | 22 | 108 | | 51.1 | | 77.1 | 3.5 | | 2.2 | | 0.63 |
| Weinberg (1991)^60^ | Centrifuged | >=5 WBC/hpf | >=10^5^ CFU/ml | | 36 | | 223 | | 5 | 755 | | 87.8 | | 77.2 | 22.4 | | 3.8 | | 0.17 |
|  |  | >=10 WBC/hpf |  |  | 32 | | 122 | | 9 | 856 | | 78.0 | | 87.5 | 23.9 | | 6.3 | | 0.25 |
| **Pyuria vs Culture (automated microscopy)** | | | | | | | | | | | | | | | | | | | |
| Armengol (2001)^29^ | Centrifuged | >=5 WBC/hpf | >=10^4^ CFU/ml | | 19 | | | 49 | 11 | | 181 | 63.3 | | 78.7 | 6.2 | | 3.0 | | 0.47 |
| Dayan (2002)^28^ | Uncentrifuged | >=5 WBC/hpf | SPA: >=10^3^, Catheter: >=10^4^ CFU/ml | | 13 | | | 13 | 7 | | 160 | 65.0 | | 92.5 | 21.4 | | 8.3 | | 0.39 |
|  |  | >=10 WBC/hpf |  |  | 9 | | | 4 | 11 | | 169 | 45.0 | | 97.7 | 31.1 | | 19.4 | | 0.56 |
| Waisman (1999)^33^ | Uncentrifuged (positives centrifuged and re-examined with standard microscopy) | >10 WBC/hpf | SPA: >=10^3^, Catheter: >=10^3^, CVU/Bag: 10^5^ CFU/ml | | 31 | | | 10 | 4 | | 76 | 88.6 | | 88.4 | 51.0 | | 7.6 | | 0.13 |
| **Baceriuruia vs Culture** | | | | | | | | | | | | | | | | | | | |
| Armengol (2000)^61^ | Not stated | Any bacteria | >=10^5^ CFU/ml | | 24 | | | 50 | 6 | | 180 | 80.0 | | 78.3 | 13.5 | | 3.7 | | 0.26 |
| Arslan (2002)^67^ | Centrifuged; Gram stain | Any bacteria | >=10^5^ CFU/ml | | 56 | | | 5 | 14 | | 25 | 80.0 | | 83.3 | 18.1 | | 4.8 | | 0.24 |
| Bulloch (2000)^32^ | Centrifuged | Any bacteria | Catheter: >10^4^, CVU: >=10^5^ CFU/ml | | 27 | | | 78 | 2 | | 52 | 93.1 | | 40.0 | 7.4 | | 1.6 | | 0.17 |
| Dayan (2000)^26^ | Late sample;  Uncentrifuged; Gram stain | Any bacteria | Catheter late stream sample: >=50000 CFU/ml. | | 6 | | | 1 | 0 | | 78 | 100.0 | | 98.7 | 680.3 | | 49.5 | | 0.07 |
|  | Late sample;  Centrifuged |  |  |  | 6 | | | 10 | 0 | | 69 | 100.0 | | 87.3 | 86.0 | | 7.1 | | 0.08 |
|  | Early sample;  Centrifuged |  |  |  | 4 | | | 16 | 2 | | 63 | 66.7 | | 79.7 | 6.9 | | 3.1 | | 0.45 |
|  | Early sample;  Uncentrifuged; Gram stain |  |  |  | 6 | | | 2 | 0 | | 77 | 100.0 | | 97.5 | 403.0 | | 29.7 | | 0.07 |
| Dayan (2002)^28^ | Uncentrifuged; Gram stain | Any bacteria | SPA: >=10^3^, Catheter: >=10^4^ CFU/ml | | 16 | | | 1 | 4 | | 172 | 80.0 | | 99.4 | 421.7 | | 138.4 | | 0.20 |
| Benito Fernandez (2000)^30^ | Uncentrifuged; Gram stain | Not stated | >50 000 CFU/ml | | 70 | | | 1 | 17 | | 87 | 80.5 | | 98.9 | 235.0 | | 70.8 | | 0.20 |
| Hiraoka (1995)^69^ | Not stated | Not stated | Catheter: >10^3^, CVU: >10^5^ CFU/ml | | 21 | | | 2 | 2 | | 64 | 91.3 | | 97.0 | 221.9 | | 30.1 | | 0.09 |
| Hoberman (1994)^71^ | Uncentrifuged; Gram stain | Any bacteria | >=50000 CFU/ml | | 95 | | | 61 | 7 | | 1975 | 93.1 | | 97.0 | 409.0 | | 31.1 | | 0.07 |
| Littlewood (1977)^78^ | Centrifuged | >=10 bacteria/hpf | >=10^5^ CFU/ml | | 33 | | | 6 | 5 | | 145 | 86.8 | | 96.0 | 136.3 | | 21.9 | | 0.14 |
| Lockhart (1995)^79^ | Uncentrifuged; Gram stain | Any bacteria | >=10^3^ CFU/ml | | 17 | | | 15 | 1 | | 174 | 94.4 | | 92.1 | 131.3 | | 11.3 | | 0.09 |
| Lohr (1993)^42^ | Centrifuged; Gram stain | Any bacteria | SPA: >10^3^, Catheter: >10^4^, CVU >10^5^ CFU/ml | | 101 | | | 170 | 1 | | 417 | 99.0 | | 71.0 | 165.7 | | 3.4 | | 0.01 |
| Matthai (1995)^38^ | Centrifuged; Unstained and Gram stain | Not stated | >=10^5^ CFU/ml | | 176 | | | 6 | 50 | | 144 | 77.9 | | 96.0 | 77.7 | | 19.5 | | 0.23 |
| Morton (1982)^17^ | Uncentrifuged | >15 bacteria/hpf | SPA: any, CVU: >10^5^ CFU/ml | | 30 | | | 22 | 38 | | 220 | 44.1 | | 90.9 | 7.8 | | 4.8 | | 0.61 |
|  | Centrifuged; Gram stain |  |  |  | 25 | | | 10 | 24 | | 200 | 51.0 | | 95.2 | 19.9 | | 10.2 | | 0.52 |
|  | Uncentrifuged; Gram stain |  |  |  | 11 | | | 2 | 10 | | 149 | 52.4 | | 98.7 | 65.5 | | 39.5 | | 0.48 |
| Purwar (1972)^80^ | Uncentrifuged; Gram stain | Any bacteria. | >=10^4^ CFU/ml | | 39 | | | 2 | 1 | | 190 | 97.5 | | 99.0 | 2007 | | 93.6 | | 0.03 |
| Pylkkanen (1979)^18^ | CVU | >=30 bacteria/hpf | SPA | | 83 | | | 5 | 116 | | 68 | 41.7 | | 93.2 | 8.9 | | 5.6 | | 0.63 |
|  |  | >1 bacteria/hpf |  |  | 172 | | | 25 | 27 | | 48 | 86.4 | | 65.8 | 11.9 | | 2.5 | | 0.21 |
| Santos (1982)^75^ | Gram stain | Not stated | Not stated | | 278 | | | 29 | 52 | | 1641 | 84.2 | | 98.3 | 295.2 | | 48.5 | | 0.16 |
| Shaw (1998)^35^ | Uncentrifuged; Gram stain | Single organism | >=10^4^ CFU/ml | | 49 | | | 45 | 13 | | 2198 | 79.0 | | 98.0 | 177.2 | | 38.8 | | 0.22 |
|  |  | Any bacteria |  |  | 50 | | | 67 | 12 | | 2176 | 80.6 | | 97.0 | 130.3 | | 27.0 | | 0.20 |
| Vangone (1985)^81^ | Uncentrifuged | >5 bacteria/ml | >=10^5^ CFU/ml | | 50 | | | 28 | 26 | | 457 | 65.8 | | 94.2 | 30.6 | | 11.4 | | 0.36 |
|  |  | >10 bacteria/ ml |  |  | 47 | | | 20 | 29 | | 465 | 61.8 | | 95.9 | 36.6 | | 14.6 | | 0.40 |
| Weinberg (1991)^60^ | Uncentrifuged; Gram stain | >=5 bacteria/oif | >=10^5^ CFU/ml | | 37 | | | 22 | 4 | | 956 | 90.2 | | 97.8 | 354.3 | | 38.8 | | 0.11 |
|  |  | >=2 bacteria/oif |  |  | 40 | | | 57 | 1 | | 921 | 97.6 | | 94.2 | 432.7 | | 16.4 | | 0.04 |
|  |  | >=1 bacteria/oif |  |  | 40 | | | 86 | 1 | | 892 | 97.6 | | 91.2 | 278.6 | | 10.9 | | 0.04 |
|  |  | Any bacteria |  |  | 40 | | | 124 | 1 | | 851 | 97.6 | | 87.3 | 184.7 | | 7.7 | | 0.03 |
| **Baceriuruia vs Culture and Microscopy** | | | | | | | | | | | | | | | | | | | |
| Vickers (1991)^82^ | Not stated | >=10^7^ bacteria /ml | Both positive | | 23 | | | 1 | 1 | | 317 | 95.8 | | 99.7 | 3316 | | 304.8 | | 0.04 |
| **Bacteriuria vs Culture (automated microscopy)** | | | | | | | | | | | | | | | | | | | |
| Armengol (2001)^29^ | Centrifuged | Any bacteria | >=10^4^ CFU/ml | | 24 | | | 50 | 6 | | 180 | 80.0 | | 78.3 | 13.5 | | 3.7 | | 0.26 |
| Manson (1985)^83^ | Diluted urine; borate buffer + acridine orange | >=10^5^ bacteria/ml | >=10^5^ CFU/ml | | 43 | | | 152 | 3 | | 1482 | 93.5 | | 90.7 | 120.8 | | 9.9 | | 0.08 |
|  | Borate buffer + acridine orange |  |  |  | 37 | | | 54 | 16 | | 1786 | 69.8 | | 97.1 | 74.5 | | 23.8 | | 0.31 |
| **Pyuria and Bacteriuria vs Culture** | | | | | | | | | | | | | | | | | | | |
| Bulloch (2000)^32^ | Centrifuged | >=5 WBC/hpf or any bacteria | Catheter: >10^4^, CVU: >=10^5^ CFU/ml | | 29 | | | 88 | 0 | | 42 | 100.0 | | 32.3 | 28.3 | | 1.5 | | 0.05 |
| Craver (1997)^36^ | Centrifuged | >10 WBC and any bacteria | Catheter: >10^3^, CVU/bag: >50 000 CFU/ml | | 17 | | | 56 | 7 | | 156 | 70.8 | | 73.6 | 6.5 | | 2.7 | | 0.40 |
| Benito Fernandez (2000)^30^ | Uncentrifuged; Gram stain | Both positive | >50 000 CFU/ml | | 81 | | | 2 | 6 | | 86 | 93.1 | | 97.7 | 433.8 | | 41.0 | | 0.07 |
|  |  | Either positive |  |  | 82 | | | 26 | 5 | | 62 | 94.3 | | 70.5 | 35.4 | | 3.2 | | 0.08 |
| Hoberman (1996)^70^ | Uncentrifuged; Gram stain | >=10 WBC/mm^3^ or any bacteria | >50 000 CFU/ml | | 203 | | | 300 | 9 | | 3741 | 95.8 | | 92.6 | 266.7 | | 12.9 | | 0.05 |
|  |  | >=10 WBC/mm^3^ and any bacteria |  |  | 186 | | | 34 | 26 | | 4007 | 87.7 | | 99.2 | 817.5 | | 104.3 | | 0.12 |
| Hoberman (1993)^84^ | Uncentrifuged; Gram stain | >=10 WBC/mm^3^ and any bacteria | >50 000 CFU/ml | | 27 | | | 2 | 5 | | 664 | 84.4 | | 99.7 | 1329 | | 281.0 | | 0.16 |
|  | Centrifuged | >=5 WBC/hpf and any bacteria |  |  | 21 | | | 5 | 11 | | 661 | 65.6 | | 99.2 | 224.9 | | 79.0 | | 0.35 |
| Hoberman (1994)^71^ | Uncentrifuged; Gram stain | >=10 WBC/mm^3^ and any bacteria | >50 000 CFU/ml | | 91 | | | 20 | 11 | | 2016 | 89.2 | | 99.0 | 782.7 | | 90.8 | | 0.11 |
| Liptak (1993)^40^ | Not stated | >=10 WBC/hpf or any bacteria | >=10^5^ CFU/ml | | 78 | | | 16 | 26 | | 209 | 75.0 | | 92.9 | 37.6 | | 10.5 | | 0.27 |
| Lohr (1993)^42^ | Centrifuged; Gram stain | >=5 WBC/hpf or any bacteria | SPA: >10^3^, Catheter: >10^4^, CVU >10^5^ CFU/ml | | 101 | | | 207 | 1 | | 382 | 99.0 | | 64.9 | 124.7 | | 2.8 | | 0.02 |
| Matthai (1995)^38^ | Centrifuged; Gram stain | Both positive: >10 WBC/hpf for pyuria not clear for others | >=10^5^ CFU/ml | | 155 | | | 3 | 71 | | 147 | 68.6 | | 98.0 | 91.7 | | 34.3 | | 0.32 |
| Shaw (1998)^35^ | Uncentrifuged; Gram stain | >=10 WBC/mm^3^ or positive gram stain. | >=10^4^ CFU/ml | | 49 | | | 314 | 3 | | 1650 | 94.2 | | 84.0 | 74.2 | | 5.9 | | 0.07 |
|  |  | >=10 WBC/mm^3^ and positive gram stain | >=10^4^ CFU/ml | | 39 | | | 20 | 13 | | 1944 | 75.0 | | 99.0 | 277.5 | | 73.7 | | 0.25 |
| Shaw (1991)^59^  *All children* | Centrifuged | >=5 WBC/hpf or few bacteria | Catheter: >=10^3^ , CVU: >=10^5^ CFU/ml. | | 43 | | | 246 | 2 | | 200 | 95.6 | | 44.8 | 14.2 | | 1.7 | | 0.12 |
|  |  | >=10 WBC/hpf and moderate bacteria | Catheter: >=10^3^ , CVU: >=10^5^ CFU/ml. | | 21 | | | 18 | 24 | | 428 | 46.7 | | 96.0 | 20.3 | | 11.6 | | 0.56 |
|  |  | >=10 WBC/hpf or moderate bacteria | Catheter: >=10^3^ , CVU: >=10^5^ CFU/ml. | | 35 | | | 85 | 10 | | 361 | 77.8 | | 80.9 | 14.3 | | 4.1 | | 0.27 |
| *<2 years* |  | >=5 WBC/hpf or few bacteria | Catheter: >=10^3^ , CVU: >=10^5^ CFU/ml. | | 12 | | | 62 | 2 | | 69 | 85.7 | | 52.7 | 5.6 | | 1.8 | | 0.32 |
|  |  | >=10 WBC/hpf and moderate bacteria | Catheter: >=10^3^ , CVU: >=10^5^ CFU/ml. | | 5 | | | 3 | 9 | | 128 | 35.7 | | 97.7 | 21.3 | | 13.8 | | 0.65 |
| **Pyuria and Bacteriuria vs Culture (automated microscopy)** | | | | | | | | | | | | | | | | | | | |
| Armengol (2001)^29^ | Centrifuged | >=5 WBC/hpf or any bacteria | >=10^4^ CFU/ml | | 27 | | | 83 | 3 | | 147 | 90.0 | | 63.9 | 13.9 | | 2.5 | | 0.16 |
| **Not stated vs Culture** | | | | | | | | | | | | | | | | | | | |
| Rodriguez Cervilla (2001)^53^ | Not stated | Not stated | SPA: >=10^2^ , Catheter: >=10^4^, CVU: >=10^5^ CFU/ml | | 36 | | | 39 | 7 | | 20 | 83.7 | | 33.9 | 2.5 | | 1.3 | | 0.50 |
| **TEST COMBINATIONS: Dipstick and Microscopy vs Culture** | | | | | | | | | | | | | | | | | | | |
| **Nitrite and pyuria** |  | |  | |  | | |  |  | |  |  | |  |  | |  | |  |
| Benito Fernandez (2000)^30^ | Either positive | | >50 000 CFU/ml | | 81 | | | 28 | 6 | | 60 | 93.1 | | 68.2 | 26.6 | | 2.9 | | 0.11 |
|  | Both positive | |  |  | 75 | | | 2 | 12 | | 86 | 86.2 | | 97.7 | 209.0 | | 30.5 | | 0.15 |
| LE, nitrite, and pyuria | | | | | | | | | | | | | | | | | | | |
| Anad (2001)^52^ | LE or nitrite and pyuria | | SPA; any; other >10 CFU/ml | | 28 | | | 50 | 6 | | 313 | 82.4 | | 86.2 | 27.2 | | 5.9 | | 0.22 |
| Bachur (2001)^85^ | Any one positive | | SPA: >=10^3^, catheter: >=10^4^, CVU: >=10^5^ CFU/ml | | 578 | | | 640 | 127 | | 7355 | 82.0 | | 92.0 | 52.1 | | 10.2 | | 0.20 |
| Lohr (1993)^42^ | LE and nitrite positive, or pyuria | | SPA: >10^3^, catheter: >10^4^, CVU >10^5^ CFU/ml | | 90 | | | 166 | 12 | | 421 | 88.2 | | 71.7 | 18.3 | | 3.1 | | 0.17 |
| **LE, nitrite and bacteriuria** | | | | | | | | | | | | | | | | | | | |
| Lohr (1993)^42^ | LE and nitrite positive, or any bacteria | | SPA: >10^3^, catheter: >10^4^, CVU >10^5^ CFU/ml | | 102 | | | 249 | 0 | | 338 | 100.0 | | 57.6 | 278.1 | | 2.3 | | 0.01 |
| **LE, nitrite, blood, and pyuria** | | | | | | | | | | | | | | | | | | | |
| Craver (1997)^36^ | All positive: LE>=trace, nitrite positive blood >=1+, and WBC>10 | | Catheter: >10^3^, CVU/bag: >50 000 CFU/ml | | 21 | | | 85 | 3 | | 127 | 87.5 | | 59.9 | 9.2 | | 2.1 | | 0.23 |
|  | All positive: LE>=trace, nitrite positive blood >=trace, and WBC>10 | |  | | 22 | | | 109 | 2 | | 103 | 91.7 | | 48.6 | 8.5 | | 1.8 | | 0.21 |
| **LE, nitrite, pyuria and bacteriuria** | | | | | | | | | | | | | | | | | | | |
| Bulloch (2000)^32^ | >=5WBC/hpf or any bacteria or >=small LE or nitrite | | Catheter: >10^4^ CFU/ml; CVU: >=10^5^ CFU/ml | | 29 | | | 87 | 0 | | 43 | 100.0 | | 33.1 | 29.3 | | 1.5 | | 0.05 |
| Lohr (1993)^42^ | Not clear | | SPA: >10^3^, catheter: >10^4^, CVU >10^5^ CFU/ml | | 102 | | | 234 | 0 | | 353 | 100.0 | | 60.1 | 309.0 | | 2.5 | | 0.01 |
| Shaw (1998)^35^ | Any one positive: >= 5 WBC/HPF or any bacteria | | >=10^4^ CFU/ml | | 79 | | | 429 | 16 | | 2870 | 83.2 | | 87.0 | 32.2 | | 6.4 | | 0.20 |
|  | Dipstick +ve and >= 5 WBC/HPF and any bacteria | |  |  | 69 | | | 66 | 26 | | 3233 | 72.6 | | 98.0 | 127.5 | | 35.9 | | 0.28 |
| Lockhart (1995)^79^ | Any positive: >=trace LE, >=5 WBC/hpf, >="slight" bacteria. | | >=10^3^ CFU/ml | | 12 | | | 40 | 6 | | 149 | 66.7 | | 78.8 | 7.1 | | 3.1 | | 0.43 |
| Arslan (2002)^67^ | Any one positive. | | >=10^5^ CFU/ml | | 52 | | | 29 | 18 | | 1 | 74.3 | | 3.3 | 0.1 | | 0.8 | | 5.38 |
