# Supplementary material for: Rapid tests and urine sampling techniques for the diagnosis of urinary tract infection (UTI) in children under five years: a systematic review
Source: BMC Pediatr. 2005 Apr 5;5:4. doi: 10.1186/1471-2431-5-4 (PMC1084351; doi:10.1186/1471-2431-5-4)
Supplement: Additional File 2 — Microsoft Word file.doc containing a table of the results of the quality assessment of included studies. [file 1471-2431-5-4-S2.doc]

## Additional Table 2 Quality assessment results for studies for the diagnosis of UTI

| **Study details** | **Spectrum composition** | **Selection criteria** | **Appropriate reference standard** | **Disease progression bias** | **Partial verification bias** | **Differential verification bias** | **Incorporation bias** | **Test execution details** | **Reference execution details** | **Test review bias** | **Diagnostic review bias** | **Clinical review bias** | **Uninterpretable results** | **Withdrawals** |
| --- | --- | --- | --- | --- | --- | --- | --- | --- | --- | --- | --- | --- | --- | --- |
| Ahmad(1991)^24^ | - | - | + | ? | + | + | + | + | + | ? | ? | ? | + | ? |
| Anad(2001)^52^ | - | + | + | + | + | + | + | + | - | ? | ? | ? | - | - |
| Armengol(2001)^29^ | - | + | + | + | + | + | + | + | + | ? | ? | ? | ? | ? |
| Armengol(2000)^61^ | ? | - | + | + | + | + | + | - | - | ? | ? | ? | ? | ? |
| Aronson(1973)^15^ | - | - | + | + | + | + | + | + | + | ? | ? | ? | ? | ? |
| Arslan(2002)^67^ | + | + | + | + | + | + | + | + | + | ? | ? | ? | ? | ? |
| Bachur(2001)^85^ | - | + | + | ? | ? | + | + | + | - | ? | ? | ? | + | + |
| Benito Fernandez(1996)^21^ | - | + | + | + | + | + | + | - | - | ? | ? | ? | + | + |
| Benito Fernandez(2000)^30^ | + | - | + | + | + | - | + | - | + | ? | ? | ? | ? | ? |
| Boreland(1986)^46^ | ? | - | + | + | + | + | + | + | + | ? | ? | ? | ? | ? |
| Braude(1967)^20^ | + | - | + | ? | + | + | + | - | + | ? | ? | ? | + | + |
| Bulloch(2000)^32^ | - | + | + | + | + | + | + | + | + | + | ? | + | ? | ? |
| Cohen(1997)^25^ | + | + | + | ? | + | + | + | + | + | ? | ? | ? | ? | ? |
| Craver(1997)^36^ | + | + | + | + | + | + | + | + | + | + | ? | ? | ? | ? |
| Dayan(2002)^28^ | - | + | + | + | + | + | + | + | + | + | + | ? | ? | - |
| Dayan(2000)^26^ | + | + | + | + | + | + | + | + | + | ? | ? | ? | + | + |
| Demi(1993)^41^ | - | - | + | ? | - | + | + | + | - | + | - | ? | ? | ? |
| Doley (2003)^66^ | - | + | + | + | + | + | + | + | + | ? | ? | ? | + | + |
| Dosa(1973)^55^ | - | - | + | + | + | - | + | + | - | ? | ? | ? | ? | ? |
| Farrell(2002)^22^ | - | + | + | + | + | + | + | + | + | ? | ? | ? | ? | - |
| Feasey(1999)^23^ | - | - | + | + | + | + | + | - | - | ? | ? | ? | + | + |
| Fennell(1977)^49^ | - | - | ? | ? | + | - | + | + | + | + | ? | ? | + | - |
| Giraldez(1998)^64^ | + | + | + | + | + | + | + | - | - | ? | ? | ? | ? | ? |
| Hardy(1976)^16^ | - | + | + | + | + | + | + | + | + | ? | ? | ? | + | ? |
| Hiraoka(1995)^69^ | + | + | + | + | + | + | + | + | + | ? | ? | ? | ? | ? |
| Hoberman(1996)^70^ | - | + | + | + | + | + | + | + | + | ? | ? | ? | ? | ? |
| Hoberman(1994)^71^ | + | + | + | + | + | + | + | + | + | + | + | ? | + | + |
| Hoberman(1993)^84^ | ? | - | + | + | + | + | + | + | + | ? | ? | ? | ? | ? |
| Holland(1968)^56^ | ? | - | + | + | + | + | + | - | - | ? | ? | ? | ? | ? |
| Kohler(1970)^57^ | - | - | + | + | + | + | + | + | - | ? | ? | ? | ? | ? |
| Kunin(1977)^50^ | - | + | + | + | + | - | + | + | + | + | ? | ? | ? | ? |
| Labbe(1982)^48^ | + | + | + | ? | + | + | + | + | + | + | - | + | ? | - |
| Lagos Zuccone (1994)^65^ | - | + | + | + | + | + | + | + | + | ? | ? | ? | ? | ? |
| Lejeune(1991)^43^ | ? | - | + | + | + | + | + | + | - | ? | ? | ? | ? | ? |
| Lin(2000)^73^ | - | + | + | + | + | + | + | + | + | ? | ? | ? | ? | + |
| Lin(2000)^72^ | - | + | + | + | + | + | + | - | + | ? | ? | ? | ? | + |
| Liptak(1993)^40^ | - | + | + | + | + | + | + | + | + | + | ? | ? | + | ? |
| Littlewood(1977)^78^ | + | + | + | + | + | + | + | + | + | ? | ? | ? | ? | ? |
| Lockhart(1995)^79^ | + | + | + | + | + | + | + | - | + | ? | ? | ? | ? | + |
| Lohr(1993)^42^ | - | + | + | + | + | + | + | + | + | ? | ? | + | ? | ? |
| Manson(1985)^83^ | - | + | + | + | + | + | + | + | + | ? | ? | ? | + | + |
| Marret(1995)^62^ | ? | - | + | + | + | + | + | + | - | ? | ? | ? | ? | ? |
| Marsik(1986)^47^ | ? | - | + | + | + | + | + | + | + | ? | ? | ? | ? | ? |
| Matthai(1995)^38^ | + | - | + | + | + | + | + | - | + | ? | ? | ? | ? | ? |
| Mendez (2003)^27^ | - | + | + | + | - | + | + | + | + | ? | ? | ? | + | + |
| Morton(1982)^17^ | + | - | + | + | + | + | + | + | + | ? | ? | ? | ? | - |
| Parmington(1989)^63^ | + | + | + | ? | + | + | + | - | - | ? | ? | ? | ? | ? |
| Pryles(1965)^74^ | ? | - | + | + | + | + | + | + | - | ? | ? | ? | ? | ? |
| Purwar(1972)^80^ | ? | - | + | ? | + | + | + | + | - | ? | ? | ? | ? | ? |
| Pylkkanen(1979)^18^ | + | - | + | + | + | + | + | - | - | ? | ? | ? | ? | ? |
| Ramage(1999)^19^ | - | - | + | + | + | + | + | + | + | ? | ? | ? | ? | ? |
| Rodriguez Caballero(2001)^68^ | ? | - | + | + | + | + | + | + | - | ? | ? | ? | + | + |
| Rodriguez Cervilla(2001)^53^ | - | + | + | + | - | + | + | - | - | ? | ? | ? | ? | ? |
| Santos(1982)^75^ | + | + | + | + | + | + | + | + | + | ? | ? | ? | ? | ? |
| Saxena(1975)^76^ | + | - | + | + | + | + | + | + | + | ? | ? | ? | ? | ? |
| Schersten(1968)^58^ | - | - | + | + | + | + | + | + | + | + | ? | ? | + | - |
| Schreiter(1971)^77^ | - | - | + | + | + | + | + | - | + | ? | ? | ? | ? | ? |
| Sharief(1998)^34^ | - | + | + | + | + | + | + | + | - | + | + | ? | + | ? |
| Shaw(1998)^35^ | + | - | + | + | + | + | + | + | + | ? | ? | ? | ? | - |
| Shaw(1991)^59^ | + | + | + | + | + | + | + | + | + | ? | ? | ? | ? | ? |
| Tahirovic(1988)^44^ | ? | - | + | + | + | + | + | + | - | ? | ? | ? | ? | ? |
| Todd(1974)^51^ | - | - | ? | ? | + | + | ? | + | - | + | ? | + | ? | ? |
| Vangone(1985)^81^ | ? | - | + | + | + | + | + | + | - | + | ? | ? | ? | ? |
| Vickers(1991)^82^ | - | + | + | + | + | + | - | + | + | ? | ? | ? | + | + |
| Villanustre Ordonez(1994)^54^ | + | - | + | + | + | + | + | + | - | ? | ? | ? | ? | ? |
| Waisman(1999)^33^ | + | + | + | + | + | + | + | + | + | ? | ? | ? | ? | ? |
| Wammanda(2000)^31^ | + | + | + | + | + | + | + | + | + | ? | ? | ? | ? | ? |
| Weinberg(1991)^60^ | - | + | + | + | + | + | + | + | + | ? | ? | + | ? | ? |
| Wiggelinkhuizen(1988)^45^ | - | - | + | ? | + | + | + | + | + | + | ? | ? | ? | ? |
| Woodward(1993)^39^ | - | + | + | ? | + | + | + | + | - | ? | ? | ? | + | ? |
